# Correction: Low-frequency transmission and persistence of antimicrobial-resistant bacteria and genes from livestock to agricultural soil and crops through compost application

**DOI:** 10.1371/journal.pone.0341735

**Published:** 2026-01-23

**Authors:** Akira Fukuda, Masato Suzuki, Kohei Makita, Masaru Usui

The images for Figs 1 and 2 are incorrect, and the image for Fig 3 is missing. The figure captions appear in the correct order. The authors have provided a corrected version of figures here.

**Fig 1:**
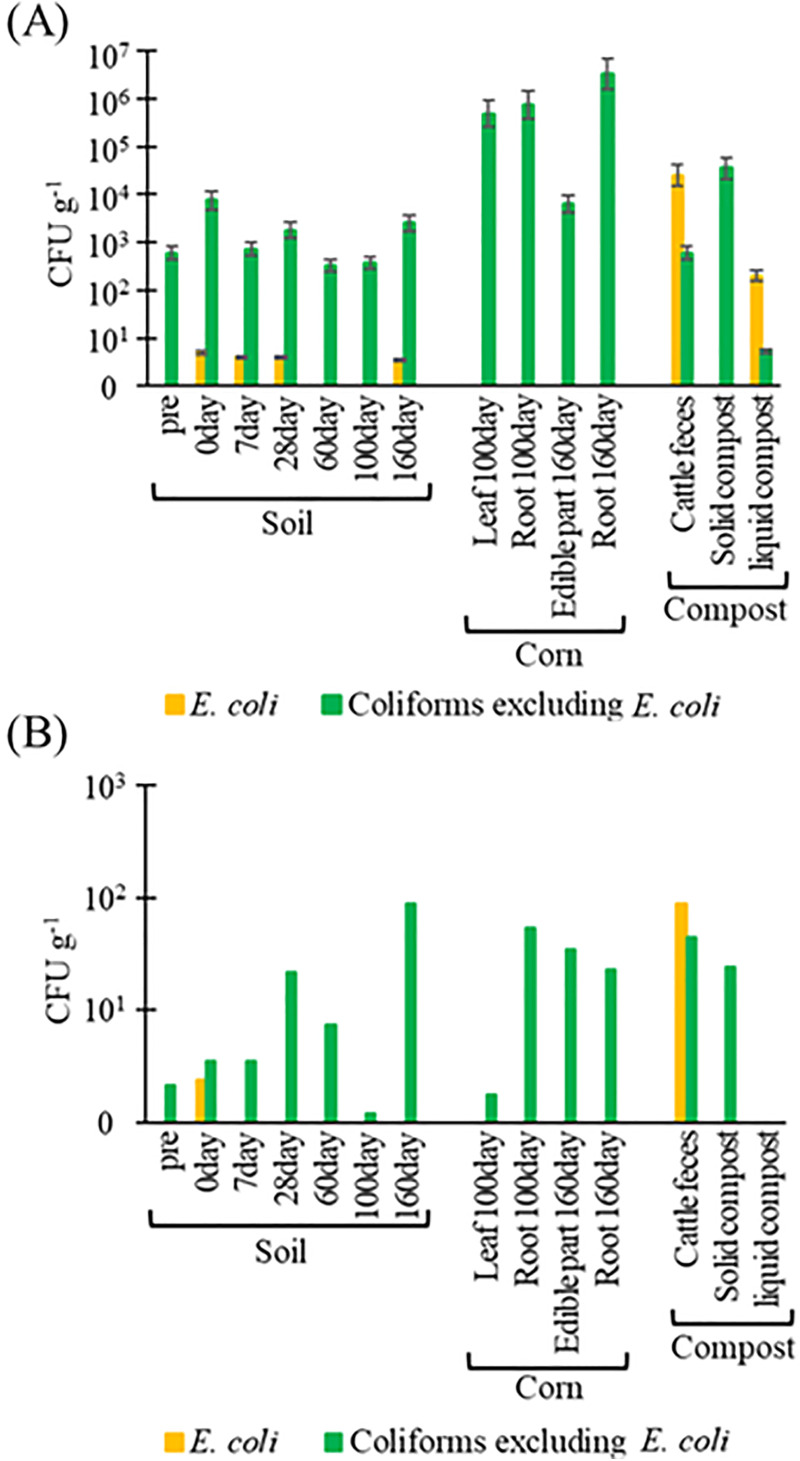
Abundance of (A) Escherichia coli and coliforms (excluding E. coli), and (B) β-lactam-resistant E. coli and β-lactam-resistant coliforms (excluding E. coli) in soil, corn, and compost. Pre: before the application of composts, day 0: day of application of compost to soils.

**Fig 2:**
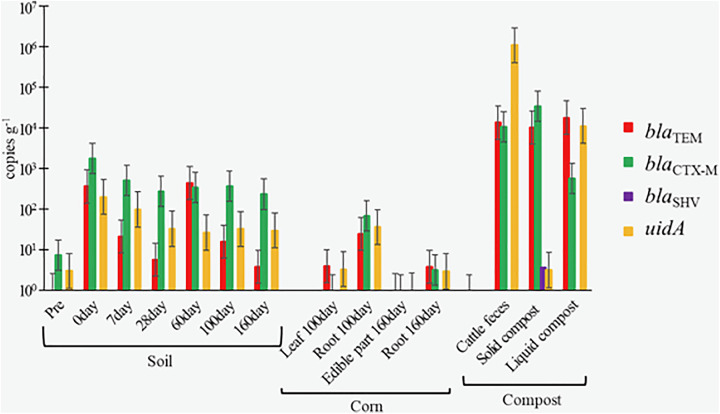
Quantification of bla and uidA genes in soil, corn, and compost via qPCR.

**Fig 3:**
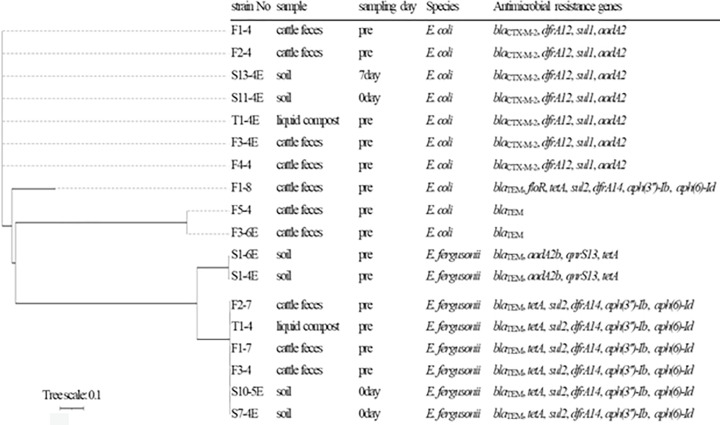
Phylogenetic tree of bla-positive β-lactam-resistant Escherichia isolates from soil, cattle feces, and compost. The phylogenetic tree was inferred from CSI phylogeny using the assembled contigs. The nodes show the strain №/source.
