# Supplementary material for: Low-frequency transmission and persistence of antimicrobial-resistant bacteria and genes from livestock to agricultural soil and crops through compost application
Source: PLoS One. 2024 May 21;19(5):e0301972. doi: 10.1371/journal.pone.0301972 (PMC11108225; doi:10.1371/journal.pone.0301972)
Supplement: Attachment — Submitted filename: Response to Reviewers.docx [file pone.0301972.s007.docx]

Author responses to reviewer comments

We are grateful to reviewers 1 and 2 for their insightful comments and suggestions, which have enriched our manuscript. As indicated in the responses below, we have taken all of these comments and suggestions into account in the revised manuscript. Additional and rephrased sentences in the main text are highlighted in yellow.

Comments to the Author

1. Is the manuscript technically sound, and do the data support the conclusions?

The manuscript must describe a technically sound piece of scientific research with data that supports the conclusions. Experiments must have been conducted rigorously, with appropriate controls, replication, and sample sizes. The conclusions must be drawn appropriately based on the data presented.

Reviewer #1: No

Reviewer #2: Partly

Response: We have rewritten the conclusion.

2. Has the statistical analysis been performed appropriately and rigorously?

Reviewer #1: No

Reviewer #2: N/A

Response: We have performed statistical analysis and described it in the Materials and Methods, and Results sections of the revised manuscript.

3. Have the authors made all data underlying the findings in their manuscript fully available?

The PLOS Data policy requires authors to make all data underlying the findings described in their manuscript fully available without restriction, with rare exception (please refer to the Data Availability Statement in the manuscript PDF file). The data should be provided as part of the manuscript or its supporting information, or deposited to a public repository. For example, in addition to summary statistics, the data points behind means, medians and variance measures should be available. If there are restrictions on publicly sharing data—e.g. participant privacy or use of data from a third party—those must be specified.

Reviewer #1: No

Reviewer #2: Yes

Response: We have added the raw data in S3 and S4 Tables.

4. Is the manuscript presented in an intelligible fashion and written in standard English?

PLOS ONE does not copyedit accepted manuscripts, so the language in submitted articles must be clear, correct, and unambiguous. Any typographical or grammatical errors should be corrected at revision, so please note any specific errors here.

Reviewer #1: Yes

Reviewer #2: Yes

5. Review Comments to the Author

Reviewer #1: 1. There are many errors from the viewpoint of science, for example, the description of HGT on Line 60-61, the use of 100 ug/mL for ampicillin resistant experiment on Line 107.

Response: In response to reviewer’s comment, we have rephrased such sentences in the revised manuscript. We used the 100 μg ml^-1^ ampicillin for the selection of β-lactam-resistant strains, as described previously (reference 17), and rephrased the sentence from “ampicillin-resistant” to “β-lactam-resistant”.

Line 62-63: Therefore, *bla* genes are among the important ARGs and should be investigated to determine the risks of dissemination

Line 105-107: without and with 100 μg mL^-1^ ampicillin (Sigma-Aldrich, St. Louis, MO, USA) to determine the abundance (CFU g^-1^) of *Escherichia coli* and coliforms (excluding *E. coli*), as well as *β*-lactam-resistant *E. coli* and coliforms (excluding *E. coli*), respectively [17].

2. The objective of this work should be more clear elaborated. For example, bla genes as the valid ARG indicator, is it fully convincable?

Response: In response to the reviewer’s comment, we rewrote the corresponding text in the revised manuscript.

Line 62-70: Therefore, *bla* genes are among the important ARGs and should be investigated to determine the risks of dissemination. To control ARB, the transmission and dynamics of ARGs must be investigated.

If composting is inadequate, residuals containing ARB and ARGs derived from livestock could spread and transmit to soils and crops. This study aimed to investigate the quantitative and qualitative transmission of livestock-derived ARB and ARGs in compost to soils and crops. First, ARB and ARG abundance was determined in cattle feces, composts, agricultural soils, and crops in the same field to evaluate the influence of compost application to agricultural soils. Second, ARB were isolated from cattle feces, composts, agricultural soils, and crops in the same field to clarify the transmission of clonal ARB isolates from livestock to soils through composts.

3. As to the isolation of bacteria, how to differentiate by their colony colors and what does it mean "from samples showing zero E.coli count" on Line 104?

Response: We apologize if our description was unclear. On CHROMagar ECC agar, colonies of *E. coli* and other coliforms showed blue and red color, respectively. In our study, homogenized samples were inoculated to CHROMagar ECC agar for quantification of *E. coli* and other coliforms. However, if they did not grow, we conducted a pre-culture step for bacterial isolation. The sentence "from samples showing zero *E. coli* count" means that colonies of *E. coli* did not grow on CHROMagar ECC agar.

Line 108-109: according to their colony colors (*E. coli*, blue colony; other coliforms, red colony)

Line 111: wherein *E. coli* colonies did not grow,

4. The biggest problem for qPCR is ambitious. Does the sample of isolates (Line 122-123) or feces/compost/soil (Line 128-129) all used? It is rather confusing.

Response: In qPCR, only field collected samples (cattle feces, solid compost, liquid compost, soil, and corn) were used, and isolates were not used. We added the sentence in the revised manuscript.

Line 135: To quantify the genes in each field sample,

5. What does it mean on Line 189-190, Line 195 "bla-positie b-lactam-resistant"?

Response: We apologize if our description was unclear and rephrase the sentence in the revised manuscript.

Line 202-203: *E. coli* was isolated from all samples using a pre-culture step, excluding the edible parts of corn (day 160) and solid composts.

Line 207-208: The *bla* genes were not detected in *β*-lactam-resistant isolates from soil (days 28, 60, 100, and 160), corn, and solid composts.

6. The section of conclusion didn't incorporate with the experimental results and data in this work. It should be improved a lot.

Response: In response to the reviewer’s comment, we have rewritten the Conclusion.

Reviewer #2: On account of the manuscript PONE-D-23-31188, entitled “Low-Frequency Transmission and Persistence of Antimicrobial-Resistant Bacteria and Genes from Livestock to Agricultural Soil and Crops through Compost Application” by Akira Fukuda et al., the authors evaluated the quantitative and qualitative transmission of livestock-derived antimicrobial resistant bacteria (ARB) and antimicrobial resistance genes (ARGs) from livestock to soil and crops through composting in Japan. The topic is important to conduct human health risk assessment for antimicrobial resistance (AMR) in the agricultural environment. After careful consideration, I feel that this manuscript is to be published after improvement of some major shortcomings. Details of my comments are as follows:

1) The view point of this research is interesting, and the authors got interesting results. Several important revisions are, however, required before publication. The first one is novelty of the research. Although the authors mentioned the aim of this study, new aspect or view point of this research was not clearly stated in the manuscript. Introduction is not well structured. The authors don’t necessarily mention general issues in detail, but are better to show information in a summarized way with focusing on the main issues related to the originality of this study. The authors are strongly encouraged to mention the novel aspects and/or viewpoints which surpass the previous researches in the manuscript clearly.

Response: In response to the reviewer’s comment, we have rewritten the Introduction.

2) The present Abstract was not informative. Abstract should include purpose of the research, principal results and major conclusions in a summarized way. In addition, due to separation of the Abstract from the major article, it must be a key to lead readers to evoke a spirit of challenge to contact with the contents of the report, as described in Author instructions of this Journal.

Response: In response to the reviewer’s comment, we have rewritten the Abstract.

3) Another notable aspect is in the experimental methodologies (validations). Although the authors mentioned the Japanese farms that were the subject of this study in Materials and Methods; Sampling locations and sample collection, the type or number of animals kept on the farms, or the specific treatment time (treatment conditions) or amount of treatment related to composting were completely missing in the manuscript. These critically affect the results of this research. The authors are encouraged to take these aspects into account to show the results with enhanced accuracy and reliability of the results.

Response: In response to the reviewer’s comment, we have added the following texts in the revised manuscript.

Line 75-87: The samples used in this study were collected from the Rakuno Gakuen University farm in Hokkaido, Japan. This encompassed a field spanning 51 000 m^2^ dedicated to the cultivation of corn. On the field, composts were applied once a year. In the Rakuno Gakuen University farm, approximately 180 dairy cattle were kept, and their excretions were treated via aerobic composting (solid compost) and anaerobic digestion (liquid compost) using a biogas plant. In aerobic composting, the temperature of the solid compost is raised by self-heating. For making a solid compost, 20% dairy cattle excretions, 50% bedding, and 30% feed residues and waste were mixed and stored at a covered, outdoor area. This mixture was turned over every 2 weeks and allowed to stand for 5 months. In anaerobic biogas digestion, liquid compost is treated at a constant temperature, removing most pathogens [16]. For making a liquid compost, 40% dairy cattle excretions, 40 bedding, and 20% farm effluent water were mixed and stored in biogas plant’s tank. In the tank, the mixture was heated to promote fermentation and kept in a month. Solid and liquid composts are kept within their designated areas on the farm. This storage continues until they are ready for use as fertilizer in the soil where corn is cultivated.

4) DNA extraction and quantitative PCR (qPCR): Since there is more than one gene region to measure for each of the bla genes (blaTEM, blaCTX-M, and blaSHV) and uidA genes that are the subject of qPCR measurements in this study, specific primer information used for the measurements, with citation of any references, need to be included in the manuscript for the accuracy and better understanding of the results.

Response: In response to the reviewer’s comment, we listed the specific primers in S2 Table.

5) Statistical processing for results and discussion for the significant difference is desirable for deepen the results and discussions. The authors are encouraged to take these aspects into account to show the results for enhancement of the novelty and better understanding of the results.

Response: We thank the reviewer for your careful assessment. We have performed statistical analysis and included this information in the Materials and Methods, and Results sections of the revised manuscript.

6) Resolutions of Figures 1A and 1B, 2, and 3 are not sufficient for publication.

Response: We have reformatted all figures to improve their resolution.
