# Supplementary material for: Low-frequency transmission and persistence of antimicrobial-resistant bacteria and genes from livestock to agricultural soil and crops through compost application
Source: PLoS One. 2024 May 21;19(5):e0301972. doi: 10.1371/journal.pone.0301972 (PMC11108225; doi:10.1371/journal.pone.0301972)
Supplement: Attachment — Submitted filename: Responses to reviewers.docx [file pone.0301972.s008.docx]

Author responses to reviewer comments

We are grateful to reviewers 1 and 2 for their insightful comments and suggestions, which have enriched our manuscript. As indicated in the responses below, we have taken all of these comments and suggestions into account in the revised manuscript. Additional and rephrased sentences in the main text are highlighted in yellow.

Reviewer #2

1. However, the "Abstract" section does not contain any of the numerical/ quantitative results of the study. It is suggested to include these results.

Response: In response to the reviewer’s comment, we have added the following texts in the revised manuscript.

Line 26-27: In the soil, 5.0 CFU g^-1^ *E. coli* was detected on day 0 (the day post-compost application), and then, *E. coli* was not quantified on 60 days post-application.

2. Critical and speculative statement needs to be incorporated in discussion regarding "why or how the burden is decrease after 7 days in soil/ compost".

Response: In response to the reviewer’s comment, we have added the following texts in the revised manuscript.

Line 245-249: Agricultural soils would be a suitable environment for fecal bacteria [25]. In the absence of antimicrobial selective pressure, it is diﬃcult for ARB to maintain dominance among the total microbes of an environment because plasmids carrying ARGs require ﬁtness costs and this causes inferior growth of ARB compared with that of the wild-type strains [30].
